# Medial augmentation plating of aseptic distal femoral nonunions

**DOI:** 10.1186/s12891-023-06675-5

**Published:** 2023-07-05

**Authors:** Sebastian Lotzien, Darius Baron, Thomas Rosteius, Charlotte Cibura, Christopher Ull, Thomas Armin Schildhauer, Jan Geßmann

**Affiliations:** 1 Department of General and Trauma Surgery, Ruhr University Bochum, Bürkle-de-La-Camp-Platz 1, 44789 Bochum, Germany; 2 Department of General and Trauma Surgery, BG University Hospital Bergmannsheil, Bürkle- de- La- Camp Platz 1, 44789 Bochum, Germany

**Keywords:** Femoral nonunion, Distal femur, Pseudarthrosis, Augmentation plating, Medial plate, Implant failure

## Abstract

**Background:**

Distal femur nonunions are well-recognized contributors to persistent functional disability, with limited data regarding their treatment options. In the current study, we asked whether additional medial augmentation plating is a feasible treatment option for patients with aseptic distal femoral nonunion and intact lateral implants.

**Methods:**

We conducted a single-center, retrospective study including 20 patients treated for aseptic distal femoral nonunion between 2002 and 2017. The treatment procedure included a medial approach to the distal femur, debridement of the nonunion site, bone grafting and medial augmentation plating utilizing a large-fragment titanium plate. Outcome measures were bone-related and functional results, measured by the Hospital for Special Surgery Knee Rating Scale (HSS) and the German Short Musculoskeletal Function Assessment questionnaire (SMFA-D).

**Results:**

Eighteen of 20 nonunions showed osseous healing at 8.16 ± 5.23 (range: 3–21) months after augmentation plating. Regarding functional results, the mean HSS score was 74.17 ± 11.12 (range: 57–87). The mean SMFA-D functional index was 47.38 ± 16.78 (range 25.74–71.32) at the last follow-up. Index procedure-associated complications included two cases of persistent nonunion and one case of infection.

**Conclusions:**

According to the assessed outcome measures, augmentation plating is a feasible treatment option, with a high proportion of patients achieving bony union and good functional outcomes and a few patients experiencing complications.

## Background

Definitive surgical interventions for distal femur fractures, which vary depending on the fracture pattern, include external fixation, open reduction and internal fixation (ORIF), intramedullary nailing (IMN) and total knee replacement [1]. Patient-related factors, e.g., diabetes and body mass index (BMI), and injury- and surgeon-associated factors, such as soft tissue status, quality of reduction and implant choice, affect outcomes [2].

The estimated incidence of nonunion after surgical intervention is approximately 5% [3, 4]. Given that distal femur nonunion is a well-recognized contributor to persistent functional disability due to persistent pain and motion deficits of the affected limb [5–8], these injuries frequently require complex revision procedures.

Few studies have compared the outcomes of distal femoral nonunion treatment [9]. Various treatment options, including revision nailing, plating and revision total knee arthroplasty, can be found in the current literature [10–12]. With respect to the evidence for a lateral femoral approach, debridement of the nonunion site with complete removal of the necrotic bone, autologous bone grafting and revision internal fixation has emerged as the most frequently used treatment option in these cases [9]. Promising results have been described for double plating of femoral nonunions, especially if the bony situation is complex [10, 13, 14]. However, in the current body of literature, the surgical approach, type of fixation and use of bone grafts have been described inconsistently.

Therefore, the purpose of this study was to analyze whether a medial approach to the femur and debridement of the nonunion site, bone grafting and additional medial augmentation plating utilizing a large-fragment titanium plate constitute a feasible treatment option for patients with aseptic distal femoral nonunion and intact lateral implants with respect to healing rates, functional outcomes and complications.

## Methods

### Study design

This single-center, retrospective study was approved by the local institutional review board (18–6269 BR). Inclusion criteria included age 18 years or older, aseptic distal femoral nonunion on admission to our hospital, an intact in situ lateral plate construct and an index procedure performed at our institution. The minimum follow-up time was twelve months.

The distal femur region was defined as the area between the distal part of the epiphysis (articular surface) and a distance of five centimeters above the femoral metaphysis. Nonunion was defined as a lack of union of the femoral fracture six months after trauma without a radiologically detectable callus bridge or at least three visible cortices on the radiographs [15]. Aseptic nonunion was defined as nonunion without clinical symptoms of infection of the affected limb, i.e., without local pain, erythema, edema, wound healing disturbance and fever and, if available, with a negative microbiological examination of intraoperative tissue cultures from the index procedure [16]. The tissue samples were incubated for 14 days.

The index procedure included a medial approach to the distal femur, complete debridement of the nonunion site, bone grafting and medial augmentation plating utilizing a large-fragment titanium plate (Synthes, Umkirch, Germany). Physical therapy was started one day after the index procedure with free knee motion. Within the first six weeks, the patients were mobilized with a maximum 15-kg weight-bearing protocol.

All patients who were treated between January 2002 and December 2017 in our department, fulfilled the inclusion criteria and were accessible via the hospital database were eligible for inclusion in this study, which consisted of a thorough follow-up of each patient’s medical history. If available, trauma radiographs were used to classify the initial pattern of the distal femoral fracture according to the Arbeitsgemeinschaft für Osteosynthesefragen and Orthopaedic Trauma Association (OTA/AO classification) [17]. The resulting nonunions were radiologically classified into atrophic and hypertrophic nonunions according to the pattern of callus formation [15]. In addition to patient demographics, clinical parameters based on the nonunion scoring system score (NUSS) were gathered [18]. Surgery-related parameters included operation time, length of hospital stay and index procedure-associated complications.

The main outcome measurements were bony healing, time to bony healing and functional outcome. According to the abovementioned definitions, bony healing was radiologically defined as the presence of bridging bone on at least three visible cortices or an evident callus bridge spanning the nonunion zone. Healing time was defined as the time between the index procedure and bony consolidation. Functional results were assessed according to the Hospital for Special Surgery Knee Rating Scale (HSS) and the German Short Musculoskeletal Function Assessment questionnaire (SMFA-D) [19, 20].

The secondary outcome measures were index procedure-associated complications, i.e., the presence of persistent femoral nonunion without any signs of ongoing osseous healing within the first twelve months after the index procedure, and infection. The definition of an index procedure-associated infection was based on the consensus definition of the AO Foundation and the European Bone and Joint Infection Society [21]. Soft tissue problems, i.e., wound breakdown and the presence of a sinus tract, in conjunction with clinical signs of infection, such as local pain, erythema, warmth and swelling in the affected limb, were counted as index procedure-associated infections regardless of whether the soft tissue samples of the index procedure tested positive for microorganisms.

The outcome measures of each patient were assessed at the final follow-up. The clinical follow-up assessments were performed at regularly scheduled visits to our outpatient clinic and included a radiographic examination with anteroposterior and lateral radiographs, a physical assessment of the affected limb and data collection to calculate the HSS score and SMFA-D score. The assessments were performed by senior consultants of the department of general and trauma surgery. Three observers independently evaluated the radiographs. In cases of uncertain status of bony consolidation, a computed tomography (CT) scan was performed to confirm or exclude bony healing.

The descriptive statistics of means and standard deviations were calculated. Data were analyzed using SPSS and Microsoft Excel.

## Results

Twenty patients with distal femoral nonunion who matched the inclusion criteria were identified and included in the study. The mean follow-up was 41.35 ± 26.90 (range: 12–103) months. The mean age of the twelve women and eight men was 59.65 ± 12.18 (range: 24–76) years. The initial fractures were classified as type A (*n* = 15), B (*n* = 1) and C fractures (*n* = 3) according to the OTA/AO classification. In one patient, initial radiographs were not available. Seventeen patients presented with atrophic nonunion, and three presented with hypertrophic nonunion on admission to our hospital. Eight of 20 patients had previously undergone 1.63 ± 1.03 (range: 1–4) revision procedures before the index procedure. The average NUSS was 33 ± 7.06 (range: 24–46).

**Fig. 1:**
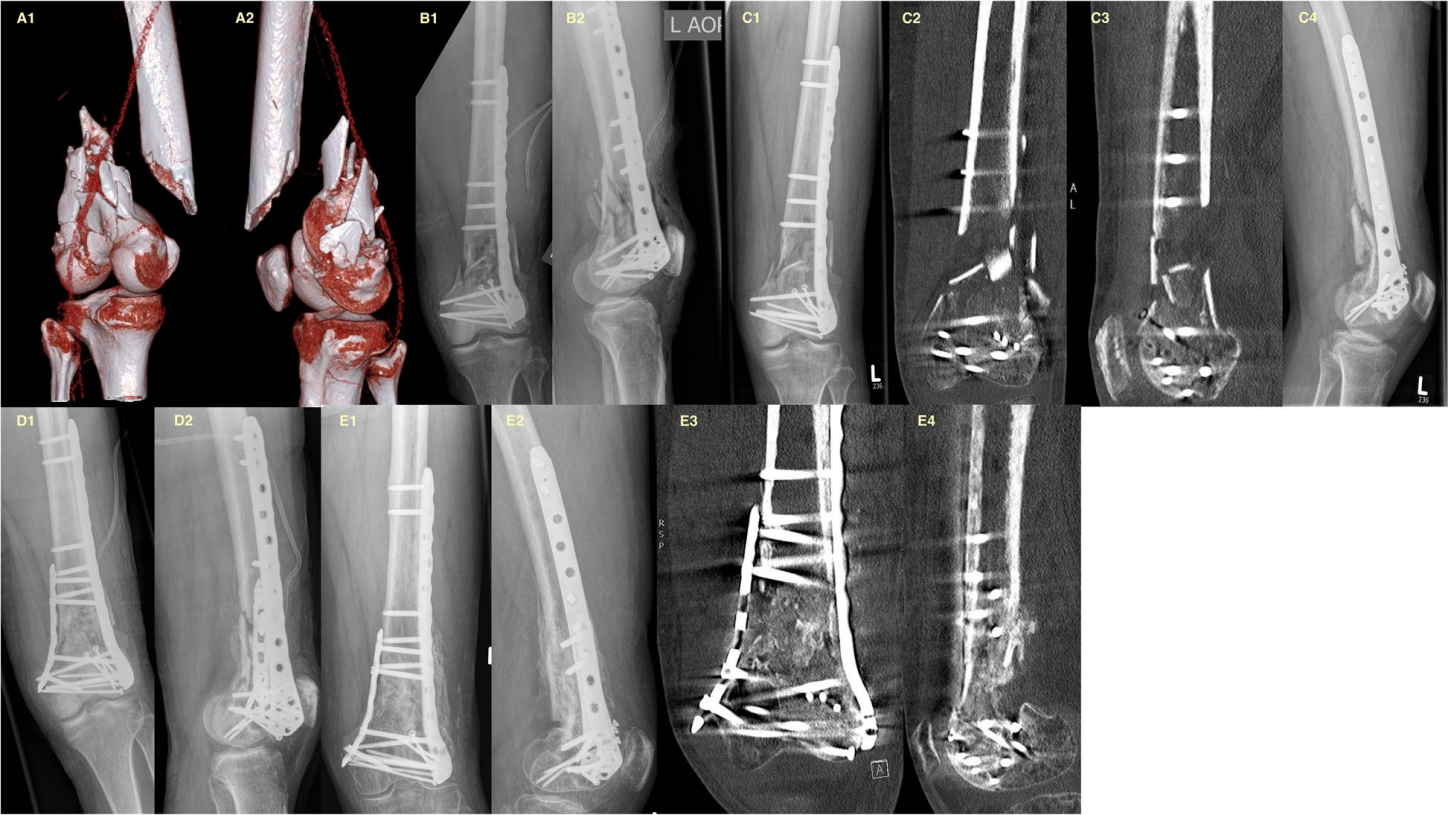
**A** Initial CT scan of an OTA type 33 – C2.3 distal femoral fracture of a 55-year-old male after a motorcycle accident. **B** X-rays showing ORIF with a lateral locking plate. **C** Nonunion six months after initial treatment. **D** Postoperative X-rays after revision utilizing a medial augmentation plate. **E** Final X-rays of the same patient showing bony healing six months after revision

A large titanium fragment plate was used in all index procedures (Fig. 1). The plate had a median of seven (range: 4 to 11) holes, and the average surgical duration was 145.05 ± 56.94 (range: 72 to 278) minutes. In 13 patients, cancellous bone grafts harvested from the anterior iliac crest were used. In five patients, the anterior crest had already been used, so the graft was taken from the posterior iliac crest. In two patients, demineralized bone matrix (Puros Allograft, Tutogen Medical GmbH, Germany) was applied instead of an autologous bone graft due to prior iliac crest usage. Additional dibotermin alfa (rhBMP-2/InductOs®) was used for four patients. In 15 of 20 patients, intraoperative tissue cultures were gathered and available for further microbiological analyses. The number of intraoperatively obtained tissue samples varied from one to three tissue samples. None of the obtained samples demonstrated bacterial growth after 14 days of incubation. Patients stayed in the hospital for 8.1 ± 6 (range: 3–32) days.

Eighteen of 20 nonunions showed osseous healing 8.16 ± 5.23 (range: 3–21) months after the index procedure. In 15 patients, CT scans were evaluated to confirm bony healing. Regarding functional results, the mean HSS score was 74.17 ± 11.12, which was rated “good”, and the range was 57–87. The mean SMFA-D functional index value was 47.38 ± 16.78 (range: 25.74–71.32), and the average bother index value was 51.19 ± 16.19 (range: 27.08–79.17).

Index procedure-associated complications included two persistent nonunions and one infection. One persistent nonunion was treated with additional bone grafting using an iliac crest bone graft, leading to bony healing six months after the revision surgery. The other patient with persistent nonunion refused further surgical revision procedures and had persistent nonunion without osseous restoration of the femur at the last follow-up. One infection was successfully treated with irrigation and debridement of the medial nonunion site in combination with empirical antibiotic therapy due to negative culture samplings despite the presence of a sinus tract and wound breakdown. Two patients with secondary arthrofibrosis of the ipsilateral knee joint underwent an arthroscopic lysis procedure during the study period (Table 1).

**Table 1:** Clinical data and classification

	**Sex**	**BMI**^**a**^	**Diabetes**	**Nicotin**	**OTA**^**b**^	**NUSS**	**Nonunion type**	**plate length (holes)**	**Bone graft**^**c**^	**Complication**	**Others**	**Final Union**	**Follow up**^**d**^
1	f	21.50	no	yes	n.a	46	atrophic	10	DBM Dibotermin			yes	17
2	m	26.27	no	no	33A3.3	42	atrophic	5	ICB		arthroscopic lysis procedure	yes	31
3	f	31.16	no	no	33A1.3	28	atrophic	8	ICB Dibotermin			yes	79
4	f	41.43	no	no	33A1.3	26	atrophic	7	ICB			yes	91
5	f	33.13	no	no	33.A	28	atrophic	11	ICB			yes	38
6	f	22.86	no	no	33A1.3	30	atrophic	6	ICB			yes	53
7	f	32.85	no	no	33C2.2	34	atrophic	8	ICB DBM	infection		yes	47
8	f	43.28	no	no	33A3	26	atrophic	5	ICB DBM			yes	38
9	m	21.95	no	no	33C3.2	28	atrophic	6	ICB			yes	73
10	m	26.73	no	yes	33C2.3	44	atrophic	6	ICB	nonunion	arthroscopic lysis procedure	yes	26
11	f	21.97	no	no	33A1.3	30	atrophic	8	ICB DBM			yes	12
12	m	32.87	yes	no	33A2.3	26	atrophic	11	DBM Dibotermin			yes	103
13	f	33.12	yes	no	33A3.2	36	atrophic	9	ICB DBM			yes	26
14	f	40.12	no	no	33A1.2	34	hypertrophic	4	ICB DBM			yes	56
15	m	40.36	no	yes	33A3.3	26	hypertrophic	7	ICB			yes	48
16	m	35.65	no	yes	33A3.1	38	atrophic	6	ICB Dibotermin			yes	19
17	m	30.07	no	yes	33A1.3	42	atrophic	8	ICB			yes	14
18	f	27.78	no	no	33B1.2	24	hypertrophic	8	ICB			yes	18
19	f	35.63	no	no	33A1.3	30	atrophic	8	ICB			yes	19
20	m	23.06	yes	yes	33A2.3	42	atrophic	8	ICB DBM	nonunion		no	19
		31.09 ± 6.66	3/20	6/20	-	33 ± 7.06				3/20		19/20	41.35 ± 26.90

^a^Body mass index (BMI = weight in kilograms divided by the square of height in meters)

^b^AO / OTA classification system

^c^*ICB* Iliac bone crest, *Dibotermin* Dibotermin alpha, *DBM* demineralized bone matrix

^d^Time in months

## Discussion

Nonunion following distal femur fracture treatment is a rare complication. Only a few studies have published reports on treatment options for these injuries and their outcomes [5, 6, 10, 11, 13, 14, 22, 23]. To our knowledge, to date, four recent studies have reported on revision double plating of distal femoral nonunions [24]. However, according to the aforementioned studies, the index procedures regularly featured a revision of the lateral in situ implant followed by re-ORIF with a double-plate construct [10, 14, 25]. Although a few studies have already reported on the results of augmentation plating of nonunions, most of the existing studies featured augmentation plating after failed fracture nailing [26, 27]. Only one recent study published by Holzmann et al. featured 16 aseptic distal femoral nonunions with intact lateral plates treated by medial augmentation plating and grafting without an additional lateral approach and revision of the in situ plates [13]. Due to the lack of current evidence, we conducted this study to report on a series of patients with distal femur nonunion and intact in situ lateral locking plate constructs treated with debridement of the nonunion zone, bone grafting and medial augmentation plating and evaluate them in terms of healing rate, functional outcomes and procedure-related complications.

**Fig. 2:**
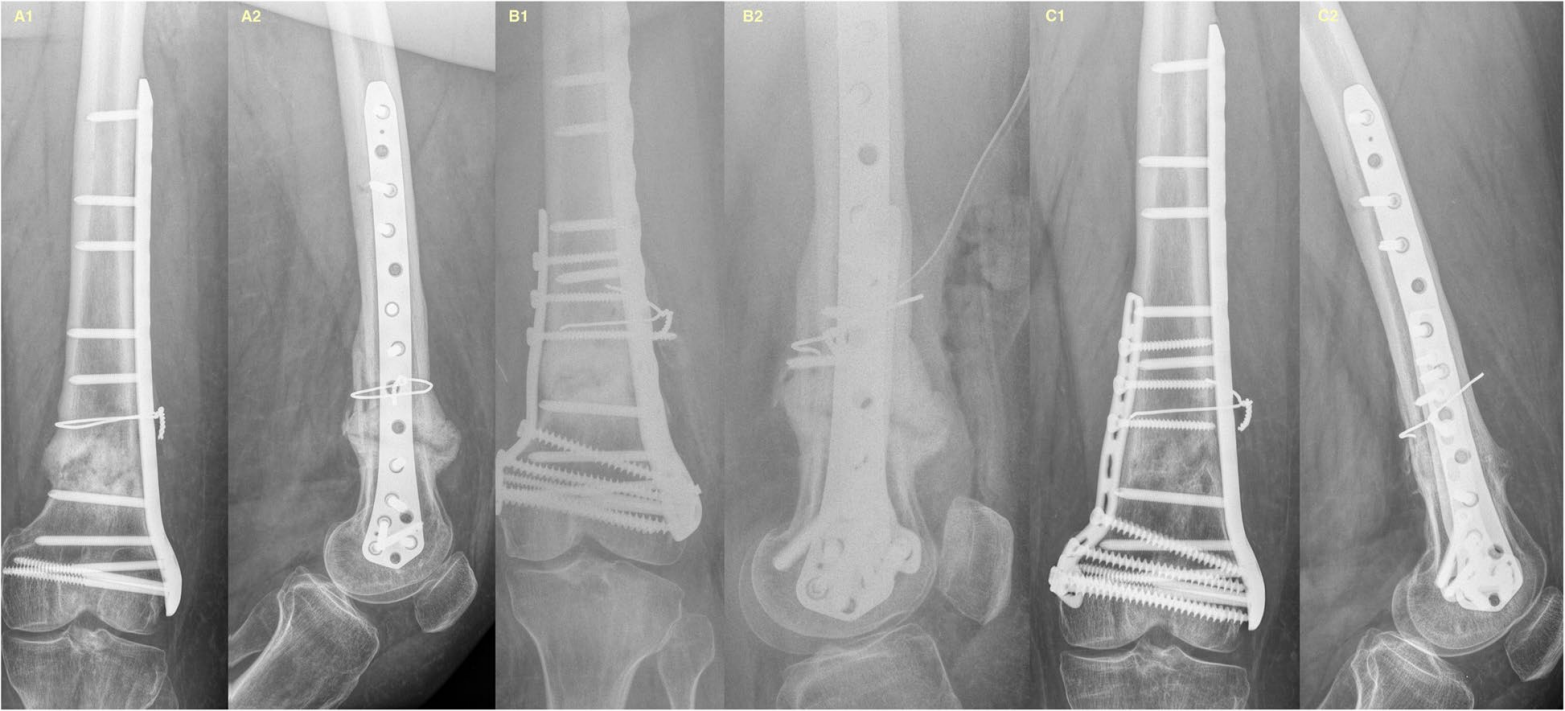
**A** Initial AP and lateral radiographs from a 24-year-old male with a BMI of 40.36 who was referred with hypertrophic nonunion with a stable in situ locking plate construct. **B** Postoperative X-rays after revision with complete debridement of the nonunion site, bone grafting and medial augmentation plating with a locking plate. **C** The patients’ final AP and lateral X-rays showing bony healing of the nonunion

In accordance with the results of this study, high union rates with bony healing rates of 90% or more have been reported for revision treatment of distal femoral nonunions [9, 28]. For instance, a retrospective study on 20 patients aged 65 years or older with supracondylar aseptic femoral nonunions treated with cement-augmented retrograde locking nails revealed a healing rate of 90% and a healing time of 4.6 months [29]. Gardner et al. reported a healing rate of 97% (30/31) in a recent series of 31 distal femoral nonunions treated with debridement of the nonunion site, bone grafting and lateral revision plating [5]. Although evidence on the optimal type of plate fixation in distal femoral fractures with respect to construct stiffness is conflicting [28, 30–32], Chapman et al. and Holzmann et al. stated that stiff double-plate constructs were superior to single-plate treatment for distal femoral nonunion, especially if the bone quality was poor [10, 13]. Chapman et al. reported a series of 18 patients with supracondylar nonunion of the femur treated with revision plate treatment and bone grafting [10]. Thirteen of the 18 patients were treated with double-plate constructs using a lateral condylar buttress plate and an additional anteromedial augmentation plate. Union was achieved in all but one patient, with an average time to union of eight months. Notably, in contrast with our study, the study of Chapman et al. included patients with fatigue failure of the in situ plates, so the nonunion was assessed using an anterolateral parapatellar approach to gain access to the lateral and anterior femur [33]. Currently, with the increasing popularity of locking plates, especially for osteoporotic bones and comminuted metaphyseal fractures of the distal femur, some femoral fractures that become nonunions show inconsistent callus formation, while an intact implant is maintained [34]. Depending on biological and construct-associated factors, i.e., the working length and plate length of the locking plate construct, both hypertrophic and atrophic nonunions can occur without implant loosening (Fig. 2) [35, 36]. Our findings emphasize that in these cases, the extended anterolateral approach is unnecessary, and the nonunion can be accurately addressed medially. Accordingly, Holzmann et al. treated 16 aseptic distal femoral nonunions with intact lateral plates by adding a medial locking plate and bone graft. Excluding their two patients who were lost to follow-up, all 14 nonunions showed union within twelve months after the intervention [13].

Although only a few studies have objectively measured functional outcomes, no important differences could be observed when comparing our outcomes with existing data [5, 6, 14, 29]. For instance, Wu et al. reported good functional results after revision nailing for 20 patients with distal femoral nonunion according to the Mize Scoring System [29, 37]. To our knowledge, the current body of evidence includes three other recent studies that have reported objective functional outcome measures after revision plating of distal femoral nonunion [5, 6, 14]. However, none of those studies included patients assessed after revision with medial augmentation plates. In brief, in accordance with our findings, all three publications indicated good functional outcomes after revision plating. However, it is plausible that the assessed SMFA-D indices in our study are lower than those of the general population, such as those published for American and Dutch populations, indicating the major impact of these injuries on quality of life and functional status [38, 39]. Notably, Kress et al. reported an average HSS score of 80.67 after treating supracondylar nonunion with associated osteoarthritis of the ipsilateral knee joint by utilizing an uncemented custom-made total knee arthroplasty with press-fit stems, indicating that this technique could serve as a salvage procedure for these complex cases [11].

During the study period, we noted three complications. In addition to the two abovementioned patients with persisting nonunion, one had an index-associated infection. Due to an epifascial infection and wound breakdown without implant loosening of the in situ double-plate construct, we decided to retain the implants in this case, considering that the femoral fascia was closed and sufficient debridement with complete resection of the necrotic soft tissue was possible. In conjunction with empirical antibiotic therapy, the infection was successfully treated, leading to consolidation of the nonunion 16 months after the index procedure. In cases with a compromised soft tissue envelope or the presence of a mature biofilm [21], the clinical approach should include the complete removal or exchange of the implants (medial and lateral), depending on the degree of bony consolidation, which we consider a potential disadvantage of our treatment algorithm. Although no vascular complications were noted during the study period, surgeons need to be aware of vascular structures traversing the medial distal femur at approximately 8 cm (descending genicular artery) and 14 cm (femoral artery) proximal to the adductor tubercle [40, 41].

Another drawback of the index procedure is the inability to correct limb deformities or shorten the femur due to the presence of an intact lateral in situ plate, so we consider the presence of limb deformities a contraindication to the medial augmentation plating and bone grafting procedure. Furthermore, two patients were treated with an additional arthrolysis procedure due to arthrofibrosis of the knee joint, but the need for this procedure was not defined as an index procedure-associated complication due to preexisting limited knee motion prior to the index procedure in these patients. Notably, the surgical duration of the index procedure averaged 145.05 min. In five patients, the bone graft was harvested from the posterior iliac crest, for which the patients were repositioned intraoperatively, leading to an average duration of the index procedure of 229 min in this subgroup.

This study has several limitations. First, due to the retrospective study design, the timespan between the index procedure and outcome assessment varied for each patient and might have induced bias. Second, the number of included patients was too small to identify patient characteristics, i.e., age, comorbidities or procedure-associated factors, that could have potentially influenced the outcome. Third, according to the surgeon’s preference and graft availability in each patient, various graft materials (i.e., iliac crest bone grafts, allografts and dibotermin alfa), which might have induced another type of bias, were used. Although bone morphogenetic proteins such as rhBMP-2 are already established methods in areas of delayed fracture healing and nonunion treatment, conflicting results on the use of growth factors have been reported in the literature and may have had an impact on the results of our study [42–47]. Fourth, due to incomplete specimen collection during the index procedure, missing data in five of the included 20 patients and the varying number of gathered tissue samples, the prevalence of a low-grade infection at the index procedure might be underestimated in our study [48]. According to the current literature, due to the high prevalence of low-grade infection in cases of nonunion and its impact on the treatment strategy, a more coherent diagnostic approach and tissue sample logistics are recommended [48, 49].

In conclusion, we have demonstrated a successful treatment option for aseptic distal femoral nonunion with intact in situ constructs. Despite the abovementioned drawbacks of the procedure, a high proportion of patients achieved bony healing with good functional outcomes and limited complication rates in our cohort.

## Data Availability

The datasets of the current study are not publicly available. Data will be available upon request to the first author, SL.

## Abbreviations

ORIF: Open reduction and internal fixation
IMN: Intramedullary nailing
BMI: Body mass index
OTA/AO: Arbeitsgemeinschaft für Osteosynthesefragen / Orthopaedic Trauma Association
NUSS: Nonunion scoring system score
HSS: Hospital for Special Surgery Knee Rating Scale
SMFA-D: German Short Musculoskeletal Function Assessment questionnaire
CT: Computed tomography
